# Factores asociados al tratamiento no exitoso para tuberculosis en pacientes previamente tratados en Cali, Colombia, en el periodo 2015-2019

**DOI:** 10.7705/biomedica.6961

**Published:** 2023-09-30

**Authors:** Liddy Varela, Jimena Ortiz, Pamela K. García, Lucy Luna, José F. Fuertes-Bucheli, Robinson Pacheco

**Affiliations:** 1 Grupo Interdisciplinario de Investigación en Epidemiología y Salud Pública, Universidad Libre, Cali, Colombia Universidad Libre Universidad Libre Cali Colombia; 2 Grupo de Investigación de Microbiología, Industria y Medio Ambiente, Universidad Santiago de Cali, Cali, Colombia Universidad Santiago de Cali Universidad Santiago de Cali Cali Colombia; 3 Semillero de Investigación de Microbiología y Salud Pública, Facultad de Ciencias de la Salud, Universidad ICESI, Cali, Colombia Universidad Icesi Universidad ICESI Cali Colombia

**Keywords:** tuberculosis, tuberculosis pulmonar, accesibilidad a los servicios de salud, cumplimiento del tratamiento, factores epidemiológicos, control de enfermedades transmisibles, Tuberculosis, tuberculosis, pulmonary, health services accessibility, treatment adherence and compliance, epidemiologic factors, communicable disease control

## Abstract

**Introducción.:**

Las tasas de éxito del tratamiento de la tuberculosis continúan siendo subóptimas.

**Objetivo.:**

Identificar los factores asociados al tratamiento no exitoso para tuberculosis en pacientes con antecedentes de tratamiento para la tuberculosis.

**Materiales y métodos.:**

Se realizó un estudio observacional retrospectivo, analítico, de cohorte de pacientes que reingresaron a un programa de micobacterias en Cali, Colombia. Se incluyeron mayores de 15 años con tuberculosis pulmonar entre el 2015 y el 2019 con antecedentes de tratamiento para la tuberculosis. Se excluyeron los pacientes con tuberculosis resistente.

**Resultados.:**

Ingresaron 605 pacientes con antecedentes de tratamiento, 60 % por tratamiento inconcluso y 40 % por recaída. En comparación con los pacientes que reingresaron por recaída (ORa= 2,34; IC=1,62-3,38), las variables que explicaron de manera independiente el no tener éxito con el tratamiento para la tuberculosis al egreso fueron: estar en situación de calle (ORa = 2,45; IC = 1,54-3,89), ser farmacodependiente (ORa = 1,95; IC=1,24-3,05), tener coinfección tuberculosis/VIH (ORa = 1,69; IC =1,00- 2,86) o diabetes (ORa =1,89; IC=1,29-2,77), y el incumplimiento de un tratamiento previo por pérdida de seguimiento, abandono u otras causas. Las variables programáticas que favorecieron el éxito del tratamiento fueron la asesoría de la prueba voluntaria de VIH (p < 0,001) y la realización de la prueba de VIH (p < 0,001).

**Conclusión.:**

Estar en situación de calle, ser farmacodependiente, tener coinfección de tuberculosis y VIH, o diabetes, así como el incumplimiento del tratamiento previo por pérdida del seguimiento, abandono o fracaso del mismo, dificultaron el éxito del tratamiento antituberculoso. En la primera atención al reingreso de los pacientes con tuberculosis se deben identificar y abordar estas características.

## Abstract (en)

**Introduction.:**

The success rates in the treatment of tuberculosis are suboptimal.

**Objective.:**

To identify associated factors with the lack of success of antituberculosis treatment in patients with a tuberculosis treatment history.

**Materials and methods.:**

We performed a retrospective, analytical, observational, and cohort study of patients reentering the Mycobacterium program in Cali, Colombia. We included patients over 15 years old with pulmonary tuberculosis between 2015 and 2019 and a history of tuberculosis treatment. Patients with drug-resistant tuberculosis were excluded.

**Results.:**

A total of 605 patients with a treatment history were included, 60% due to unfinished treatment and 40% due to relapse. Compared to patients reentering due to relapse (ORa=2.34, CI=1.62-3.38), the independent variables associated with treatment failure at discharge were homelessness (ORa=2.45, CI=1.54-3.89), substance dependence (ORa=1.95, CI=1.24-3.05), tuberculosis/HIV coinfection (ORa=1.69, CI=1.00-2.86), diabetes (ORa=1.89, CI=1.29-2.77), and unfinished previous tuberculosis treatment due to follow-up loss, abandonment, or other causes. Programmatic variables favoring treatment success were voluntary HIV testing counseling (*p*<0.001) and HIV testing (*p*<0.001).

**Conclusion.:**

Homelessness, substance dependence, tuberculosis/HIV coinfection, diabetes, and incomplete previous treatment due to loss to follow-up, abandonment, or treatment failure hindered the success of antituberculosis. These characteristics should be identified and addressed during the initial care of patients reentering treatment for tuberculosis.

La tuberculosis es una enfermedad infectocontagiosa producida por especies del complejo *Mycobacterium tuberculosis* que puede afectar cualquier tejido, principalmente los pulmones ^1^. La tuberculosis presenta una distribución mundial y afecta principalmente a personas en estado de inmunosupresión o con vulnerabilidades sociales, tales como pobreza extrema, hacinamiento, población privada de la libertad, marginados sociales, personas en situación de calle y desplazados ^2^.

Según la Organización Mundial de la Salud (OMS), se estima que 10,6 millones de personas se enfermaron de tuberculosis en el 2021. La tasa de incidencia de nuevos casos de tuberculosis por 100.000 personas por año aumentó un 3,6 % entre el 2020 y el 2021, lo que posicionó a esta enfermedad -incluso durante la pandemia por COVID-19- como la infección con mayor mortalidad por un agente infeccioso único. Si bien hubo una reducción en los casos recién diagnosticados también se presentó un aumento de la mortalidad, lo que indica un probable subregistro y un abordaje deficiente durante la pandemia por COVID-19 ^2^^-4)^. En Colombia, la tasa de incidencia de todas las formas de tuberculosis para el 2021 fue de 25,9 casos por 100.000 habitantes, donde las entidades territoriales con las mayores tasas fueron: Amazonas, Risaralda, Meta, Barranquilla, Cali, Guaviare y Arauca ^5^.

A pesar de los esfuerzos, la incidencia y la carga de la enfermedad se mantiene resistente al tratamiento principalmente en los países de bajos ingresos y en áreas de exclusión social de los países de medianos ingresos. La estrategia mundial “Fin de la tuberculosis” tiene las metas necesarias para disminuir las implicaciones en salud, económicas y sociales causadas directa e indirectamente por la tuberculosis ^2^^,^^6^. Sin embargo, al ritmo actual no se lograrán dichas metas en la región, especialmente en los países con los índices más bajos de desarrollo humano y producto interno bruto ^7^.

Además de otras intervenciones, para el control de la tuberculosis es necesario lograr el éxito en el tratamiento, ya que los pacientes con antecedentes de incumplimiento del tratamiento o recaída de la infección tienen mayor riesgo de presentar tuberculosis resistente y multirresistente ^8^^-^^10^. Sin embargo, el éxito terapéutico puede dificultarse por las características sociodemográficas de la población como sexo, edad, condición económica, vulnerabilidad, etnia y, en Colombia, tener seguro de salud subsidiado o no tener ningún seguro. Esto puede conllevar a la pérdida del seguimiento o abandono del mismo, también por dificultades en la atención de los pacientes y sus horarios laborales, los eventos adversos de los medicamentos, redes de apoyo frágiles, dificultades en la relación médico-paciente, educación deficiente, ausencia de recordatorios y falta de uso de tecnologías adaptadas culturalmente ^11^^-^^16^, así como la falta de esquemas terapéuticos estandarizados que evalúen la sensibilidad a los medicamentos ^17^.

En el 2020, las tasas de éxito del tratamiento contra la tuberculosis a nivel mundial fueron del 86 %, pero fue menor para pacientes con tuberculosis resistente y para aquellos de regiones con bajos recursos ^2^^-^^4^^,^^18^. En Colombia, para este mismo año, se reportaron 12.582 casos de tuberculosis, de los cuales el 84,8 % correspondió a tuberculosis pulmonar. El 90,6 % fueron casos nuevos o recaídas, mientras que el 9,4 % fueron casos previamente tratados. En Cali se reportaron 1.102 casos: el 86,5 % fueron nuevos o recaídas, y el 13,5 % casos previamente tratados ^19^. El éxito del tratamiento de la tuberculosis en Colombia -entre el 2009 y 2019- no ha superado el 75,3 % ^5^.

Debido a que la tasa de éxito puede variar de acuerdo con las características de la población ^20^^,^^21^ y a otros determinantes sociales de la salud, se precisa que los programas encaminados al tratamiento y manejo de esta enfermedad evalúen las condiciones de reingreso y egreso de los pacientes, e identifiquen los indicadores programáticos que se ven afectados entre los pacientes que recayeron o no finalizaron el tratamiento previo por alguna razón ^11^^-^^13^.

Por lo tanto, este estudio buscó identificar las diferencias clínicas, sociodemográficas y programáticas en pacientes previamente tratados para tuberculosis que reingresaron en un programa de identificación, diagnóstico y tratamiento de la tuberculosis durante el periodo 2015-2019. Se identificaron los factores asociados al tratamiento no exitoso para tuberculosis.

## Materiales y métodos

Se llevó a cabo una investigación operativa mediante un estudio observacional descriptivo de una cohorte retrospectiva de pacientes que reingresaron al Programa de Micobacterias de Santiago de Cali en las categorías de recaída y tratamiento previo inconcluso, según la clasificación de la Resolución 227 de 2020 ^6^ y las definiciones operativas recomendadas por la Organización Mundial de la Salud (OMS) ^22^^,^^23^, basadas en la historia de tratamiento previo de tuberculosis.

Se compararon los grupos de reingreso: I) tratamiento previo inconcluso, es decir, pérdida del seguimiento, al tratamiento no exitoso para tuberculosis y otros pacientes previamente tratados no clasificados como recaída, y II) recaída, o sea, pacientes previamente tratados por tuberculosis, declarados curados o con tratamiento completo al final del último ciclo y ahora diagnosticados con un episodio recurrente de tuberculosis, ya sea una verdadera recaída o un nuevo episodio de tuberculosis causado por reinfección.

### Criterios de selección

Se incluyeron registros de sujetos mayores de 15 años con antecedentes de tratamiento para tuberculosis y que al ingreso a un programa de micobacterias tenían una prueba bacteriológica positiva para tuberculosis pulmonar (cultivo, baciloscopia, prueba molecular o todos), entre el 2015 y el 2019. Se excluyeron registros sin información completa para la variable de desenlace, menores de 15 años y pacientes con tuberculosis multirresistente, debido a que las definiciones y abordajes recomendados para estas poblaciones son diferentes ^22^^,^^23^.

### Población de estudio

El estudio se realizó en Cali (Colombia), organizada en 22 comunas y con una población estimada de 2’252.616 habitantes para el 2020 ^24^. Se seleccionó y se analizó la información de los pacientes mayores de 15 años que reingresaron al Programa de Micobacterias entre el 2015 y el 2019 y que tenían antecedentes de tratamiento para tuberculosis, bien sea por tratamiento previo incompleto o por recaída.

Para las variables de desenlace, se usaron las definiciones de resultados del tratamiento de pacientes con tuberculosis de la OMS ^22^^,^^23^. En la variable de desenlace de interés se tomó el “tratamiento no exitoso” en la que se incluyeron todos los casos no exitosos del tratamiento para tuberculosis, fallecidos por tuberculosis y perdidos en el seguimiento; y el “tratamiento exitoso”, es decir, que terminaron el tratamiento como curados o con tratamiento completo culminado con evidencia de prueba negativa. Los casos fatales de tuberculosis que se incluyeron fueron los que la Secretaría de Salud clasificó como fallecidos por tuberculosis de acuerdo con las unidades de análisis, mientras que los casos fatales por otras causas fueron excluidos de los análisis que hace el Programa de Micobacterias.

### Fuentes de la información

Toda la información se obtuvo de la base de datos del Programa de Micobacterias de la Secretaría de Salud de Cali, reportados en el libro de pacientes con tuberculosis ante el Sistema de Vigilancia de Salud Pública (Sivigila). Se analizaron las variables sociodemográficas y clínicas contenidas en la fuente de información.

### Análisis estadístico

La información se almacenó en una hoja de Microsoft Excel® y se analizó con el programa Stata^™^, versión 16 (Stata Corp, Collage Station, TX, USA). Las características de la población de estudio fueron resumidas con estadística descriptiva. La distribución normal de los datos de las variables numéricas se determinó mediante la prueba de Shapiro-Wilk y se tomaron como valores significativos aquellos menores o iguales al valor de p (p ≤ 0,05). Las variables numéricas se resumieron en medianas y rangos intercuartílicos, mientras que las variables cualitativas se expresaron como proporciones y se presentan en tablas de frecuencias.

Mediante un análisis bivariado y con tablas de 2 x 2 se evaluó la asociación entre las variables de exposición (clínicas, demográficas y programáticas) con el desenlace de interés (tratamiento no exitoso para tuberculosis al egreso). Para determinar la magnitud de la asociación se calcularon las razones de probabilidad (*odds ratio*, OR) con su respectivo intervalo de confianza (IC) del 95 %. Para evaluar la significancia estadística se aplicaron las pruebas de ji al cuadrado y U de Mann-Whitney según correspondiera.

Para determinar el peso de cada variable de exposición en el desenlace, y ajustar por posibles variables de confusión, se realizó un análisis multivariado usando una regresión logística múltiple. La capacidad del modelo saturado se calculó con el 10 % de los casos que fracasaronj (-1), como lo sugiere Silva y Barroso ^25^. Las variables para la construcción de este primer modelo se seleccionaron con una significancia estadística de p ≤ 0,25 en el análisis bivariado como lo sugiere Hosmer y Lemeshow ^26^ y mediante la aproximación estadística *backwards* se eligió el modelo más parsimonioso según la prueba de verosimilitudes. La sensibilidad y la especificidad del modelo final para clasificar correctamente las observaciones se hizo por medio de un análisis de curvas ROC.

### Aspectos éticos

En esta investigación no hubo ninguna intervención ni modificación de las variables de los individuos. Este estudio fue clasificado por el Comité de Investigación Humana de la Universidad Libre, Seccional Cali, como “sin riesgo” de acuerdo con la Resolución 8430 de 1993 del Ministerio de Salud de Colombia.

## Resultados

**Figura 1:**
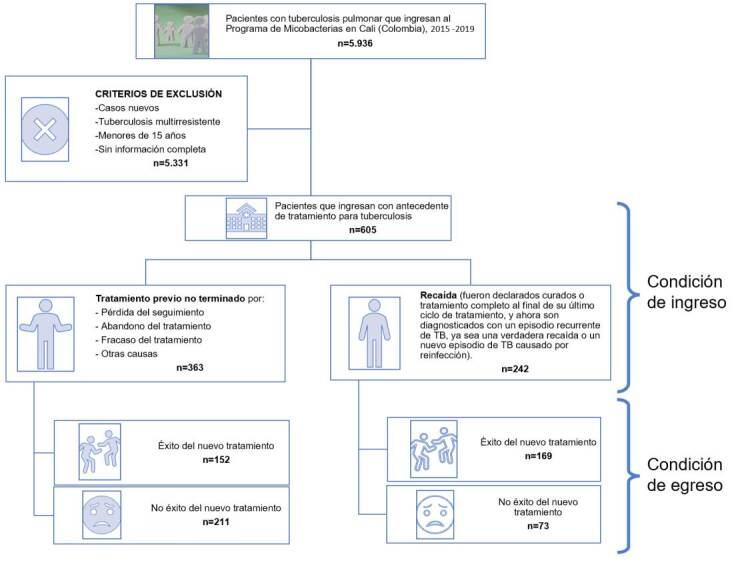
Diagrama de flujo de la población de pacientes con tuberculosis pulmonar previamente tratados en Cali, según su condición de ingreso y egreso, 2015-2019

**Figura 2:**
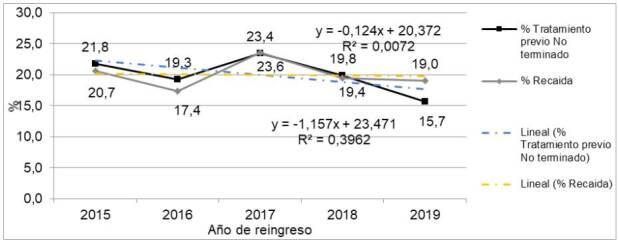
Frecuencia del tratamiento previo no terminado y recaída al tratamiento farmacológico de registros con tuberculosis pulmonar previamente tratados en Cali entre el 2015 y el 2019

Entre enero del 2015 y diciembre del 2019, ingresaron 605 sujetos al Programa de Micobacterias, de los cuales 363 (60 %) tenían antecedente de tratamiento previo inconcluso: 38 (10,5 %) estaban clasificados como tratamiento farmacológico no exitoso para tuberculosis, 12 (3,3 %) abandonaron el tratamiento y 313 (86,2 %) se habían perdido en el seguimiento. Los 242 (40 %) pacientes restantes reingresaron al programa en la categoría de recaídas (figura 1). El comportamiento temporal fue estable para ambas cohortes y ninguna de las diferencias superó los cuatro puntos porcentuales (figura 2).

La mediana de la edad de la población fue de 43 años (RI = 17-96), el sexo masculino fue el más frecuente (71,4 %) y los indígenas y afrodescendientes no fueron predominantes. En cuanto a la Seguridad Social en Salud, sólo dos de cada diez pertenecían al régimen contributivo. La coinfección de tuberculosis y HIV se reportó en el 12,4 %. Otras comorbilidades fueron reportadas en el 43,9 %, con las cardiometabólicas -diabetes (68,4 %) e hipertensión (31,9 %)- como las más frecuentes. Con relación al diagnóstico, el 67,9 % reportó una baciloscopia positiva y al 87,4 % se les realizó asesoría y prueba voluntaria para HIV (cuadro 1).

**Cuadro 1:** Diferencias clínicas y sociodemográficas de pacientes previamente tratados para tuberculosis pulmonar que reingresaron al Programa de Micobacterias de Cali, 2015-2019

Variables clínicas y sociodemográficas		Antecedentes de tratamiento previo no terminado (n=363)	Recaída (n=242)	p
Edad (años) Mediana		39	49	0,020*
		RI: 29 - 53	RI: 39 - 60	
Sexo				0,880
	Femenino	103	70	
	Masculino	260	172	
Pertenece a etnia indígena				0,350
	Sí	4	1	
	No	359	241	
Pertenece a etnia afrodescendiente				0,290
	Sí	69	38	
	No	294	204	
Pertenece a otra etnia				0,210
	Sí	290	203	
	No	73	39	
Régimen de seguridad social en salud				0,001
	Contributivo	60	67	
	No contributivo	303	175	
Población desplazada				0,810
	Sí	2	1	
	No	361	241	
Persona en situación de calle				<0,001
	Sí	98	21	
	No	265	221	
Población farmacodependiente				0,001
	Sí	88	32	
	No	275	210	
Población gestante				0,240
	Sí	2	0	
	No	361	242	
Población privada de la libertad				0,001
	Sí	18	31	
	No	345	211	
Resultado de la baciloscopia				0,800
	Positivo	248	163	
	Negativo	115	79	
Asesoría para la prueba de VIH				0,060
	Sí	310	219	
	No	53	23	
Prueba voluntaria de HIV				0,200
	Sí	325	224	
	No	38	18	
Coinfección de tuberculosis-HIV				0,130
	Sí	51	24	
	No	312	218	
Comorbilidades				0,780
	Sí	205	108	
	No	158	134	
Diabetes				<0,001
	Sí	142	49	
	No	221	193	
Hipertensión				<0,001
	Sí	142	51	
	No	221	191	
Comorbilidad no clasificada				0,070
	Si	17	20	
	No	346	222	

RI: rango intercuartílico

Al evaluar las condiciones sociodemográficas y clínicas al ingreso al programa, se encontraron diferencias estadísticamente significativas en la edad. El grupo de recaídas fue mayor que el grupo con antecedentes de tratamiento previo incompleto (mediana=49; RI=39-60 versus mediana=39; RI=29-53). El pertenecer al régimen no contributivo (grupo conformado por las personas sin seguridad social y personas subsidiadas) fue más frecuentemente reportado en el grupo de tratamiento previo incompleto (p = 0,001), al igual que estar en situación de calle (p < 0,001), ser población farmacodependiente (p = 0,001) o privada de la libertad (p = 0,001). Además, el grupo con antecedentes de tratamiento previo inconcluso presentó mayor proporción de diabetes (p < 0,001) e hipertensión (p < 0,001).

De los pacientes que reingresaron, el 58,1 % de aquellos con antecedentes de tratamiento previo incompleto fracasó con el nuevo tratamiento, y el 30,2 % de los pacientes con antecedentes de recaída (cuadro 2). Al evaluar los factores asociados con el tratamiento no exitoso para tuberculosis en los pacientes previamente tratados, se identificaron asociaciones estadísticamente significativas en las condiciones sociales, principalmente las que connotan vulnerabilidad social -personas en situación de calle y farmacodependientes- (cuadro 2)-, mientras que la asesoría para la prueba voluntaria de HIV y su realización fueron factores que favorecieron el éxito del tratamiento. Además, el reingreso con antecedentes de tratamiento previo inconcluso aumentó en 2,21 veces el riesgo de no tener éxito con el nuevo tratamiento, en comparación con reingresar con antecedentes de recaída y tener diabetes, hipertensión o coinfección de tuberculosis/HIV (cuadro 2).

**Cuadro 2:** Análisis bivariado para identificar factores relacionados con el fracaso del tratamiento antituberculoso en pacientes que reingresaron al Programa de Micobacterias de Cali, 2015-2019

Características		Tratamiento		OR	IC_95 %_	p
		No exitoso (n=284)	Exitoso (n=321)			
Mediana de edad (años)						
	< 42	155	147	1,42	1,01-1,98	0,031
	≥ 43*	129	174			
Sexo						
	Masculino	211	221	1,3	0,90-1,89	0,13
	Femenino*	73	100			
Pertenece a etnia indígena						
	Sí	2	3	0,75	0,06-6,6	0,75
	No*	282	318			
Pertenece a etnia afrodescendiente						
	Sí	48	59	0,9	0,57-1,40	0,63
	No*	236	262			
Régimen de seguridad social en salud						
	Contributivo	45	82	0,54	0,35-0,83	0,003
	No contributivo*	239	239			
Población desplazada						
	Sí	0	3	--	--	--
	No*	284	318			
Persona en situación de calle						
	Sí	80	39	2,83	1,82-4,44	<0,001
	No*	204	282			
Población farmacodependiente						
	Sí	72	48	1,93	1,26-2,96	0,001
	No*	212	273			
Población gestante						
	Sí	0	2	--	--	--
	No*	284	319			
Población privada de la libertad						
	Sí	19	30	0,69	0,36-1,31	0,23
	No*	265	291			
Resultado de la baciloscopia						
	Positivo	200	211	1,24	0,86-1,77	0,21
	Negativo*	84	110			
Asesoría prueba voluntaria de HIV						
	Sí	232	297	0,36	0,20-0,61	<0,001
	No*	52	24			
Se realizó prueba de HIV						
	Sí	245	304	0,35	0,18-0,65	<0,001
	No*	39	17			
Coinfección tuberculosis-HIV						
	Sí	44	31	1,61	1,02- 2,90	0,02
	No*	240	290			
Cualquier comorbilidad						
	Sí	123	143	0,95	0,68-1,32	0,75
	No*	161	178			
Diabetes						
	Sí	120	71	2,57	1,78-3,73	<0,001
	No*	164	250			
Hipertensión						
	Sí	120	73	2,48	1,72-3,59	<0,001
	No*	164	248			
Condición de ingreso al programa						
	Antecedentes de tratamiento previo no terminado	211	152	3,21	2,24-4,60	
	Recaída*	73	169			

OR: *odds ratio*

* categoría de referencia

El análisis multivariado permitió identificar que estar en condición de calle (*Odds Ratio* ajustado, ORa = 2,45; IC_95 %_ = 1,54-3,89), ser farmacodependiente (ORa = 1,95; IC_95%_ = 1,24-3,03), ingresar con antecedente de tratamiento previo incompleto (ORa = 2,34; IC_95%_ = 1,623,38), tener coinfección de tuberculosis/VIH (ORa = 1,69; IC_95%_ = 1,00-2,86) o diabetes (ORa = 1,89; IC_95%_ = 1,29-2,77) explicaron de manera independiente al tratamiento no exitoso para tuberculosis con antecedentes de tratamiento para tuberculosis. Solo dos variables con significancia estadística menor a 0,05 en el análisis bivariado (régimen de seguridad social y la hipertensión) fueron excluidas del modelo final (cuadro 3). El desempeño del modelo propuesto para explicar el tratamiento no exitoso para tuberculosis a través del conjunto de variables presentadas en el cuadro 2 reportó que el 66,7 % de los datos fue clasificado correctamente.

**Cuadro 3:** Análisis multivariado para identificar factores relacionados al fracaso del tratamiento antituberculoso en pacientes que reingresaron al programa de Micobacterias de Cali, 2015-2019.

Características	Tratamiento		ORc IC_95 %_	ORa IC_95 %_	p
	No exitoso (n=284)	Exitoso (n=321)			
Persona en situación de calle					
Sí	80	39	2,83	2,83	<0,001
No*	204	282	1,82-4,44	1,82-4,44	
Población farmacodependiente					
Sí	72	48	1,93	1,93	0,003
No*	212	273	1,26-2,96	1,26-2,96	
Coinfección tuberculosis-HIV					
Sí	44	31	1,71	1,71	0,049
No*	240	290	1,02-2,9	1,02-2,9	
Diabetes					
Sí	120	71	2,57	2,57	0,001
No*	164	250	1,78-3,73	1,78-3,73	
Condición de ingreso al programa Antecedentes de tratamiento previo no terminado	211	152	3,21	3,21	<0,001
Recaída*	73	169	2,24-4,60	2,24-4,60	

ORc: *odds ratio* crudo; ORa: *odds ratio* ajustado; IC: intervalo de confianza

* Categoría de referencia

## Discusión

Este estudio describe las características demográficas, clínicas y programáticas de los pacientes con tuberculosis con antecedente de tratamiento previo que reingresaron a un programa de identificación, diagnóstico y tratamiento de tuberculosis en Cali - por tratamiento inconcluso o recaída - y expone los factores relacionados con tratamiento no exitoso para tuberculosis. El 58,1 % de los pacientes que reingresaron con antecedente de tratamiento previo incompleto y el 30,2 % de los pacientes que reingresaron por recaída presentaron un tratamiento no exitoso. Los factores que explicaron de manera independiente la menor probabilidad de éxito al tratamiento en los pacientes con antecedente de tratamiento para tuberculosis fueron cinco: estar en situación de calle, ser farmacodependiente, tener coinfección de tuberculosis/VIH o diabetes y reingresar con antecedente de tratamiento previo no terminado; mientras que recibir asesoría para la prueba de VIH voluntaria y hacerse la prueba para este virus, favorecieron el éxito del tratamiento.

La frecuencia de pacientes con tuberculosis previamente tratada que reingresaron al programa fue del 10,2 %. Esta proporción de pacientes está entre el porcentaje reportado para Colombia en el 2019 y el reportado para Perú en el 2021 ^19^^,^^27^. Por otra parte, la OMS en su informe del 2020 de tuberculosis en las Américas reportó que, en la región, el éxito del tratamiento en los retratamientos fue del 44,0 %, sin incluir los casos de recaídas. En esta investigación se encontró que el éxito del tratamiento para los pacientes con tratamiento previo inconcluso fue levemente menor (41,9 %). Para el caso de las recaídas, la OMS reportó un éxito del tratamiento del 75,6 % ^28^, resultado similar a lo observado en este estudio, donde casi siete de cada diez pacientes que ingresaron por recaída tuvieron resultados exitosos del tratamiento.

El éxito del tratamiento de la tuberculosis en Colombia no ha superado el 75,3 %, y el comportamiento de la mortalidad por tuberculosis en las Américas no ha presentado el descenso esperado para alcanzar las metas de la estrategia mundial “Fin de la tuberculosis” ^7^. Entre los pacientes que reingresaron por tratamiento previo inconcluso o por recaída, fueron muy pocos los que pertenecían al Régimen de Seguridad Social en Salud Contributivo, pues la mayoría presentaba algún factor de vulnerabilidad. Estos hallazgos han sido ampliamente documentados por otras investigaciones, convirtiendo el abordaje de los determinantes sociales de la salud como un pilar fundamental para el control de la tuberculosis ^7^^,^^18^. Por esta razón, los sistemas de salud deberían incluir a estos grupos vulnerables en el diseño de estrategias innovadoras para el seguimiento estrecho del tratamiento y el abordaje de otros determinantes sociales.

Con frecuencia, los hombres presentan menores probabilidades de éxito con el tratamiento, lo cual se ha relacionado con un menor cumplimiento ^29^. Sin embargo, en nuestro estudio no hubo asociación entre el sexo y el reingreso al programa, al igual que en otro estudio en Colombia ^30^. No obstante, se evidenció que el principal determinante para no tener éxito con el tratamiento antituberculoso actual fue el tener historia de tratamiento previo inconcluso por pérdida de seguimiento o problemas de cumplimiento o de abandono del tratamiento, a diferencia de los que reingresaron por recaída.

El estar en situación de calle aumentó la probabilidad de tratamiento no exitoso para tuberculosis en 2,45 veces. En Brasil, estar sin hogar aumentó el riesgo del tratamiento no exitoso para tuberculosis en 2,38 veces ^31^, y se ha identificado que la población sin hogar presenta una tasa de pérdida durante el seguimiento 2,9 veces mayor ^32^. Por otra parte, este estudio reporta que ser farmacodependiente aumenta la probabilidad de tratamiento no exitoso para tuberculosis en 1,95 veces.

Otras investigaciones han publicado resultados similares y han referido que el consumo de sustancias, especialmente inyectadas, puede disminuir el cumplimiento del tratamiento y favorecer las pérdidas en el seguimiento ^33^^,^^34^, comportamiento similar al señalado por el estudio en Brasil de Soares *et al*., 2001-2014 ^35^, en el que la mayoría de las personas con antecedente de consumo de algún psicoactivo interrumpió el tratamiento antituberculoso. Las características propias de los pacientes en situación de calle o de farmacodependientes pueden dificultar el cumplimiento del tratamiento y el seguimiento, por lo que en esta población podría ser beneficioso el abordaje multidisciplinario y el uso de estrategias novedosas de seguimiento ^11^^-^^14^.

El tener coinfección de tuberculosis y HIV también aumentó la probabilidad del tratamiento no exitoso para tuberculosis en 1,69 veces. En África se han reportado resultados similares ^18^^,^^36^, ya que la coinfección con VIH generó un riesgo de tratamientos fallidos de 1,53 veces ^18^. Además, en este estudio el tener diabetes aumentó la probabilidad de fracasar en el tratamiento en 1,89 veces. Un estudio en Armenia (Colombia) de pacientes con tuberculosis, independientemente de su estado de ingreso, reportó que la probabilidad de fracasar con el tratamiento fue 8,99 veces mayor en aquellos con diabetes que en aquellos sin diabetes ^37^. Estos resultados pueden deberse, entre otras razones, a la polifarmacia o al abordaje integral deficiente dadas las características de fragmentación y segmentación del sistema de salud en Colombia.

El ingresar al programa con antecedente de tratamiento previo inconcluso aumentó la probabilidad de tratamiento no exitoso para tuberculosis 2,34 veces, en comparación con el ingresar por recaída. En Etiopía se ha reportado una probabilidad de falla del tratamiento de 5,32 veces ^38^. Además, el antecedente de tuberculosis se asocia con un cumplimiento deficiente del tratamiento ^39^, y en África, el retratamiento aumentó el riesgo de resultados fallidos en 1,48 veces ^18^.

Este hallazgo puede indicar que no se están abordando efectivamente las características de los pacientes que contribuyeron al tratamiento previo no exitoso para tuberculosis y que permiten la recurrencia de la pérdida en el seguimiento y del abandono del tratamiento. Ante este panorama se hace necesario documentar estas características al ingreso de los pacientes en los programas de tuberculosis, ya que puede permitir la identificación de aquellos con menores posibilidades de lograr el éxito del tratamiento e intervenirlos rápidamente con estrategias que garanticen el seguimiento estrecho y el cumplimiento del tratamiento.

En cuanto a las estrategias que podrían aumentar el éxito del tratamiento, en este estudio se identificó que el recibir asesoría para la prueba voluntaria del HIV y el hacérsela, lo favorecieron. Previamente se ha reportado como útil la educación y el asesoramiento del paciente, los incentivos y los facilitadores, las intervenciones psicológicas, los recordatorios y los rastreadores, y las tecnologías de salud digital ^40^. Los hallazgos de la presente investigación pueden explicarse por la mayor cercanía e información entregada al paciente por parte del personal de salud durante la asesoría y la ejecución de la prueba de HIV. Además, como eran intervenciones voluntarias, los resultados también pueden deberse al compromiso e interés de los pacientes por el tratamiento de su enfermedad. La educación del paciente suele ser subvalorada, factor que puede favorecer la circulación de conceptos erróneos que dificultan la participación de los pacientes en su tratamiento y afectan sus decisiones ^41^^,^^42^. Por lo tanto, el ofrecer información clara, oportuna y comprensible del porqué y para qué del tratamiento ^43^^,^^44^ y el adaptarla según las características propias del paciente ^45^, puede favorecer el éxito del tratamiento y promover comportamientos saludables.

La principal limitación de este estudio fue su naturaleza retrospectiva de fuentes secundarias, lo cual puede comprometer la calidad de la información. Sin embargo, la cantidad de información analizada incluyó la totalidad de los registros disponibles, así como la amplitud del periodo a un quinquenio. La amplitud del periodo evaluado connota variabilidad en el sistema de vigilancia acompañada de posibles sesgos de selección y clasificación. Por esta razón, algunas definiciones programáticas fueron estandarizadas por los investigadores haciendo uso de los manuales y los protocolos del Instituto Nacional de Salud de Colombia ^5^^,^^6^.

Dado que esta es una investigación operativa, la generalización de los resultados debe tomarse con cautela. En general, estas limitaciones resaltan la necesidad de interpretar los resultados de este estudio con precaución y de considerarlos como una parte de la evidencia disponible relacionada con el tema de estudio. Se requieren investigaciones adicionales con diseños más rigurosos y controlados para obtener conclusiones más sólidas.

En conclusión, el estar en situación de calle, ser farmacodependiente, tener coinfección de tuberculosis-HIV o diabetes, así como haber recibido tratamiento previo para tuberculosis y reingresar por tratamiento previo no terminado (pérdida en el seguimiento, problemas de cumplimiento o abandono del tratamiento), se asoció con el fracaso del nuevo tratamiento.

Por lo tanto, se recomienda identificar estas características en la población que reingresa a los programas de tuberculosis y acrecentar la captación, el tratamiento y el seguimiento estrecho de los pacientes, junto con otras estrategias que garanticen el éxito del tratamiento y mejoren su cumplimiento, como la educación del paciente y la promoción de su autocuidado.

Además, se recomienda diferenciar a los pacientes con antecedentes de tratamiento previo inconcluso o recaída, ya que en los primeros es más probable la recurrencia del tratamiento no exitoso para tuberculosis.

Esta investigación proporciona información para diseñar intervenciones dirigidas a lograr las metas de éxito del tratamiento de la tuberculosis y disminuir el riesgo de la tuberculosis resistente.
